# Noise Suppression for GPR Data Based on SVD of Window-Length-Optimized Hankel Matrix

**DOI:** 10.3390/s19173807

**Published:** 2019-09-03

**Authors:** Wei Xue, Yan Luo, Yue Yang, Yujin Huang

**Affiliations:** 1 School of Automation, China University of Geosciences, Wuhan 430074, China; 2 Hubei Key Laboratory of Advanced Control and Intelligent Automation for Complex Systems, Wuhan 430074, China

**Keywords:** ground-penetrating radar, noise suppression, singular value decomposition, Hankel matrix, window length optimization

## Abstract

Ground-penetrating radar (GPR) is an effective tool for subsurface detection. Due to the influence of the environment and equipment, the echoes of GPR contain significant noise. In order to suppress noise for GPR data, a method based on singular value decomposition (SVD) of a window-length-optimized Hankel matrix is proposed in this paper. First, SVD is applied to decompose the Hankel matrix of the original data, and the fourth root of the fourth central moment of singular values is used to optimize the window length of the Hankel matrix. Then, the difference spectrum of singular values is used to construct a threshold, which is used to distinguish between components of effective signals and components of noise. Finally, the Hankel matrix is reconstructed with singular values corresponding to effective signals to suppress noise, and the denoised data are recovered from the reconstructed Hankel matrix. The effectiveness of the proposed method is verified with both synthetic and field measurements. The experimental results show that the proposed method can effectively improve noise removal performance under different detection scenarios.

## 1. Introduction

Ground-penetrating radar (GPR) is a geophysical detecting instrument that transmits high-frequency electromagnetic wave and receives the reflections [1]. GPR has been widely used in several fields such as civil engineering, archaeology, geology, and military exploration [2,3,4,5,6] for its nondestructive, continuous, rapid, and efficient properties. Due to the effect of complex underground environment [7] and ultra-wide bandwidth receiver [8], the echoes of GPR contain significant noise. The noise collected by the system can easily mask the effective signals. Therefore, noise suppression is very important for improving the signal quality and interpretation accuracy.

Different approaches for GPR noise suppression have been reported to the literature [9,10,11,12,13,14,15,16,17,18,19,20,21]. The wavelet transform is a popular method for GPR data denoising [9,10], and it is simple and effective. However, the selection of the mother wavelet function, the decomposition level, and the threshold function still rely on subjective experiences. Frequency-wavenumber (F-K) filtering originating from seismic data denoising has also been applied to remove noise in GPR data [11,12] and can remove cross rebar reflections and ringing noise effectively. However, the filter design in the F-K domain is relatively complex and the method is only suitable for point targets. The ensemble empirical mode decomposition (EEMD) method is an improved empirical mode decomposition (EMD) method carrying out the EMD over an ensemble of the signal plus Gaussian white noise. The EEMD method can extract the effective signals components from noisy GPR data [13,14]. However, the EEMD method is time-consuming and incapable of processing the raw data with a low signal-to-noise ratio (SNR). The robust principle component analysis (RPCA) method can recover a low-rank matrix from noisy measurements and it has been employed to suppress the clutter and noise of GPR data [15,16,17]. However, the RPCA method is sensitive to the choice of thresholds. Singular value decomposition (SVD) is a convenient method to decompose a matrix, which can decompose GPR data into different subspaces that correspond to different components [18,19,20,21]. The noise can be suppressed by selecting components that contain effective signals to reconstruct GPR signals. Since each component corresponds to one singular value, the key problem of denoising is the selection of appropriate singular values corresponding to effective signals. A criterion based on the SNR of recovered data has been applied for GPR signal denoising [22], which shows better performance than the wavelet threshold denoising method. The local energy ratio rule has been used to remove background noise of GPR signals [23], which exhibits good robustness under different detection conditions. The fuzzy c-means (FCM) clustering rule has been used to extract multiple targets in heavily cluttered GPR images [24], which can accurately separate the overlapping boundaries of clutter, noise, and target signals and improve the performance of conventional SVD. 

Although the denoising methods based on SVD are effective and easy to implement, they are designed to decompose a matrix (two-dimensional data) and cannot fully separate effective signals from the noise in one-dimensional data. To resolve this problem, the one-dimensional data can be transformed into many kinds of matrices, such as the Toeplitz matrix, cycle matrix, and Hankel matrix. The difference lies in the method of creating the matrix, which will affect signal processing of SVD. Among the matrices, SVD of the Hankel matrix can achieve a similar signal processing effect to the wavelet transform [25]. Therefore, SVD of the Hankel matrix is more suitable for noise suppression. A scheme based on SVD of the Hankel matrix has been used to reduce noise for radar cross-section (RCS) data [26], which can improve the accuracy of target recognition greatly. The Hankel matrix-based SVD can eliminate the false peak in processing an impulse signal with strong trend and enhance the SNR in the reconstructed signal [27], which helps to improve the fault diagnosis performance for rolling bearings. The SVD and Hankel matrix-based denoising process has also been applied to the ball bearing vibration signals in both time and frequency domain for the elimination of the background noise [28]. It was found that denoising in the frequency domain yields better fault identification results than the denoising in the time domain. The SVD method based on the Hankel matrix in the local frequency domain has been applied to eliminate random noise in GPR data [29], which can improve suppression of random noise around non-horizontal phase reflection events.

Although the aforementioned papers have proven the effectiveness of SVD of the Hankel matrix in noise suppression, little research has been conducted with respect to the influence of the Hankel matrix size on denoising performance. The size of the Hankel matrix depends on the length of the sliding window which affects the information quantity that can be extracted from this matrix [30]. Based on this previous research, this paper proposes SVD of a window-length-optimized Hankel matrix to suppress noise for GPR data. First, the Hankel matrix formed by one-dimensional GPR data is decomposed with SVD, and the fourth root of the fourth central moment (FRFCM) of singular values is used to select the optimal window length of the Hankel matrix. Then, one threshold is generated by the difference spectrum of singular values, which is used to select effective signal components. Finally, the Hankel matrix is reconstructed with singular values corresponding to effective signals to suppress noise, and the denoised data are recovered from the reconstructed Hankel matrix. The performance of the proposed method is verified with series of synthetic and field measurements. The experimental results of the proposed method are also compared with those of the conventional SVD method based on the local energy ratio rule and wavelet transform method. The results show that the proposed method can effectively improve the denoising performance for GPR data.

## 2. Methodology

### 2.1. Denoising Method Based on SVD of the Hankel Matrix

The two-dimensional GPR data can be denoted by $B\in R^{N\times L}$, where *L* is the number of traces and *N* is the number of sampling points in each trace. For the data of one trace (one-dimensional data) *X*=[x(1),x(2),…,x(*N*)], a Hankel matrix can be formed by sliding a window over the corresponding vector [25], which can be written as

(1) $$A=\left[\begin{array}{cccc} x(1) & x(2) & \cdots & x(n)\\ x(2) & x(3) & \cdots & x(n+1)\\ \vdots & \vdots & \vdots & \vdots \\ x(m) & x(m+1) & \cdots & x(N) \end{array}\right]$$

where m=N−n+1, 1<n≤m<N, $A\in R^{m\times n}$, and n is the window length.

The SVD of Hankel matrix A can be expressed as

(2) $$A=USV^{T}$$

where $U\in R^{m\times m}$ and $V\in R^{n\times n}$ are the left singular and right singular orthogonal matrices, respectively. $S=diag\left(\sigma_{1},\sigma_{2},\ldots ,\sigma_{r}\right)$ is a singular value matrix with $\sigma_{1}\geq \sigma_{2}\geq \ldots \geq \sigma_{r}\geq 0$, and r=min(m,n). According to the definition of Equation (1), the number of singular values r is equal to the window length n.

Then, Equation (1) can be written as

(3) $$A=\sum_{i=1}^{r}\sigma_{i}u_{i}v_{i}^{T}=\sum_{i=1}^{n}\sigma_{i}u_{i}v_{i}^{T}$$

where $u_{i}\in R^{m\times 1}$ and $v_{i}\in R^{n\times 1}$. $u_{i}v_{i}^{T}\in R^{m\times n}$ is the single rank matrix, which is the *i*th eigen image of *A*. It is obvious that $\sigma_{i}$ is actually the projection of matrix *A* on the basis $u_{i}v_{i}^{T}$.

As singular values are arranged in descending order, the first few larger singular values generally correspond to effective signals with strong correlations, while the smaller singular values correspond to the noise with weak correlation. Therefore, matrix *A* can be written as

(4) $$A=\sum_{i=1}^{k}\sigma_{i}u_{i}v_{i}^{T}+\sum_{i=k+1}^{n}\sigma_{i}u_{i}v_{i}^{T}$$

where k is the demarcation point of singular values, and the first k singular values correspond to effective signals? Then the Hankel matrix with noise suppression can be reconstructed as

(5) $$A_{s}=\sum_{i=1}^{k}\sigma_{i}u_{i}v_{i}^{T}$$

According to the construction rule of the Hankel matrix, the denoised one-dimensional data can be given by

(6) $$X_{s}=\left[A_{S}(1,:),\;A_{S}(2:m,n)\right]$$

where $A_{S}(1,:)$ is the first row of matrix $A_{S}$ and $A_{S}(2:m,n)$ is the last column without the first element.

### 2.2. Optimization Method of Window Length

The window length n is the only parameter of the Hankel matrix which not only affects the information quantity extracted from the matrix but also the performance of SVD. As an example, synthetic one-dimensional GPR data are used to analyze the effect of the window length n on the performance of SVD. The synthetic data are generated by the “gprMax” simulator [31].

**Figure 1:**
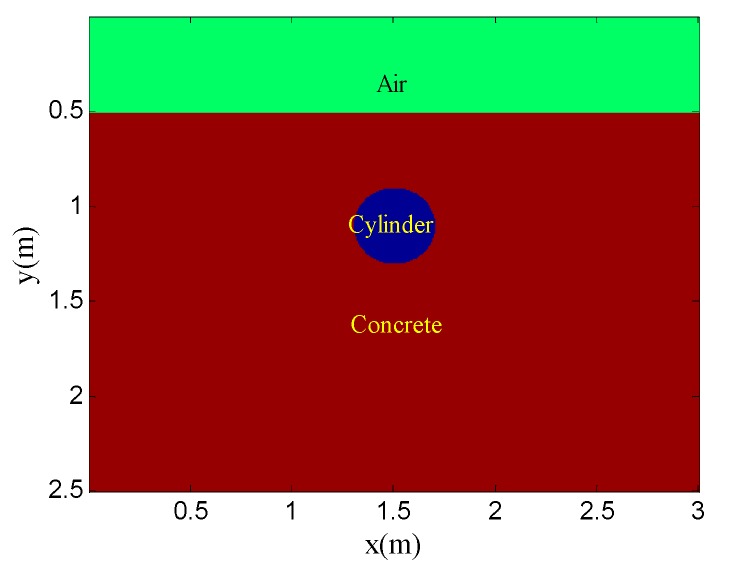
Geometry of the simulation model for point target detection.

Figure 1 shows the geometry of the simulation model for the scenario. The background medium is concrete. The relative permittivity and conductivity are 6 and 0.01, respectively. The target is a perfect metal cylinder, with 0.4-m diameter, which is buried at a depth of 0.6 m. The Ricker wavelet with a center frequency of 900 MHz is adopted. There are 80 traces in total and the trace interval is 0.035 m. The time window for each trace is 12 ns and each trace contains 2036 sampling points.

**Figure 2:**
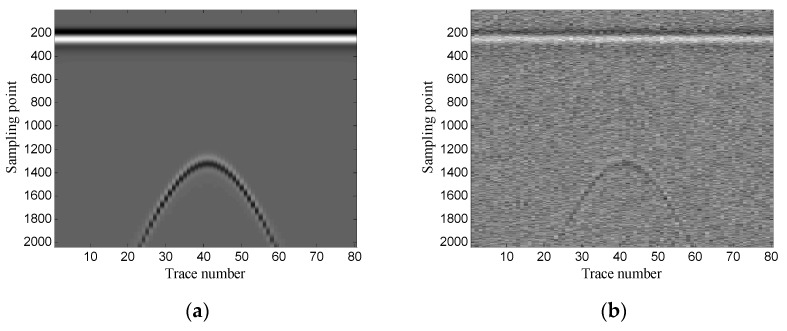
Synthetic ground-penetrating radar (GPR) image: (**a**) original image; (**b**) noisy image.

**Figure 3:**
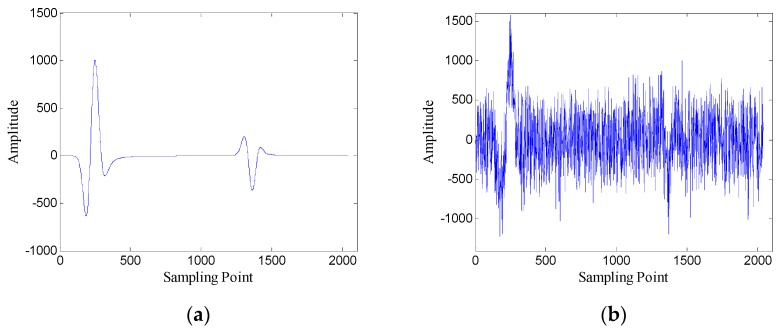
Data of trace 38: (**a**) original data; (**b**) noisy data.

The Gaussian white noise is added to the original GPR image and the SNR is −5.00 dB. Figure 2 shows the original GPR image and the noisy GPR image. Figure 3 shows the original data and noisy data of trace 38. The direct wave and target echoes are near the 250th and the 1300th sampling points, respectively. The noisy data are used to form the Hankel matrices with different window lengths, and SVD is applied to the Hankel matrices. 

**Figure 4:**
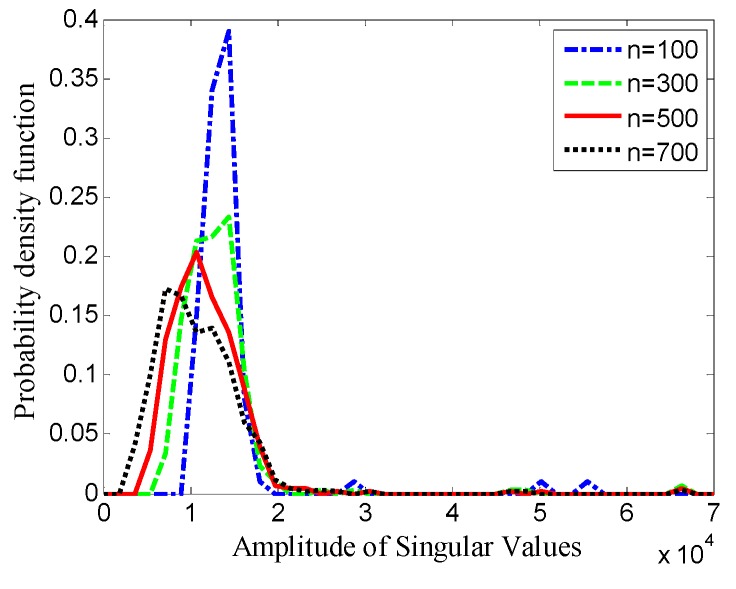
Probability distribution of singular values for Hankel matrices with different window lengths.

Figure 4 shows the probability distribution of singular values for Hankel matrices with different window lengths. The few larger singular values corresponding to effective signals are distributed in a relatively wide range, and the distribution is sparse. However, the smaller singular values corresponding to the noise are distributed in a narrow range, and the distribution is approximately normal. Moreover, the window length has an obvious effect on the probability distribution of singular values.

For noise suppression, when the distance between the distribution of singular values corresponding to effective signals and the distribution of singular values corresponding to the noise increases, it is easier to distinguish between effective signal components and noise components, which helps to improve noise removal performance. Based on the analysis of distribution characteristics of singular values in Figure 4, the fourth root of the fourth central moment (FRFCM) of singular values is proposed to measure the distance between the two distributions, which is defined by

(7) $$P(n)={\left(\frac{1}{n}\sum_{i=1}^{n}{\left(\sigma_{i}-\bar{\sigma }\right)}^{4}\right)}^{\frac{1}{4}}$$

where n is the number of singular values, $\sigma_{i}$ is the *i*th singular value, and $\bar{\sigma }$ is the mean of singular values. 

In order to obtain optimal noise suppression performance, P(n) should be maximized. Therefore, the optimal window length can be given by

(8) $$n_{opt}=\arg\underset{n}{\max}\left[P(n)\right]$$

### 2.3. Selection Method of Singular Values 

The number of singular values selected results in a trade-off between noise suppression and recovery of the signal of interest. The selection methods based on SNR of recovered data [22] and local energy ratio [23] merely consider the energy of singular values, and their performance degrades when the SNR is relatively low. The selection method based on FCM clustering [24] uses a membership function to find suitable singular values corresponding to effective signals, which is relatively complex.

**Figure 5:**
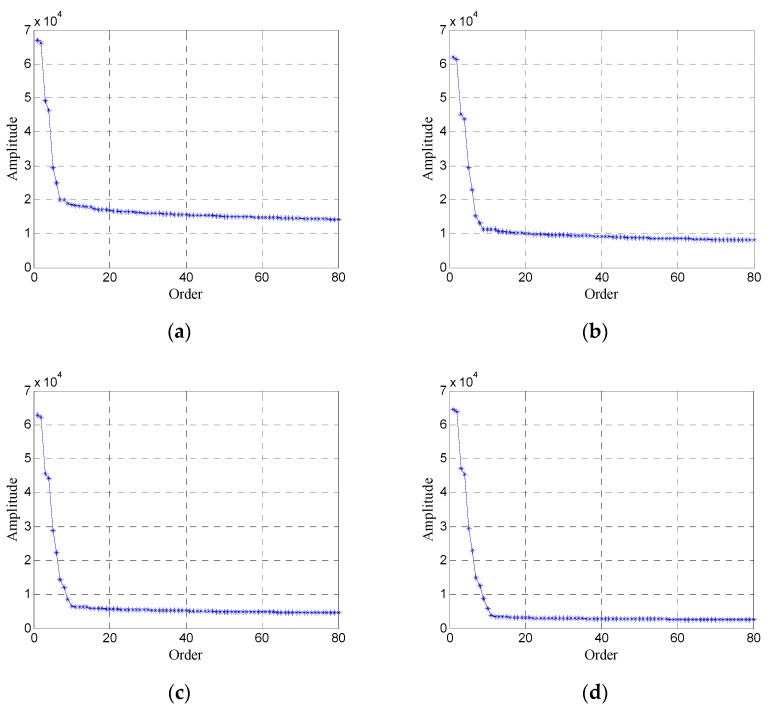
Variation of singular values for Hankel matrices under different signal-to-noise ratios (SNRs): (**a**) SNR = −5 dB; (**b**) SNR = 0 dB; (**c**) SNR = 5 dB; (**d**) SNR = 10 dB.

In order to obtain an efficient and accurate selection of singular values, the synthetic data in Section 2.2 are used to analyze the variation of singular values. Figure 3 shows the variation of singular values for Hankel matrices (the window length is 300) under different SNRs. For simplicity, only the first 80 singular values are shown in Figure 5.

As shown in Figure 5, the first few singular values correspond to effective signals, and they are larger and decrease quickly with the increase of order; the remaining singular values correspond to the noise, and they are smaller and decrease slowly with the increase of order. For noise, when the SNR increases, the amplitude of singular values decreases obviously and the number of singular values also decreases slightly. For effective signals, when the SNR increases, the amplitude of singular values changes little and the number of singular values increases slightly.

Based on the analysis of variation characteristics of singular values, the difference spectrum of singular values is used to find the demarcation point between singular values corresponding to effective signals and singular values corresponding to the noise. The difference spectrum of singular values [32] can be defined as

(9) $$b_{i}=\sigma_{i}-\sigma_{i+1}\;\;\;i=1,2,\cdots r-1$$

where $\sigma_{i}$ is the *i*th singular value and r is the number of singular values.

The mean of the difference spectrum of singular values is calculated, and a threshold is given by

(10) $$T=\frac{\rho }{r-1}\sum_{i=1}^{r-1}b_{i}$$

where ρ is a weight coefficient that adjusts the threshold.

Then, the threshold *T* is used to select singular values corresponding to effective signals. To improve the accuracy of the selection, three adjacent difference spectra are compared with the threshold *T* to obtain the demarcation point

(11) $$k_{1}=i|\;b_{i}<T\;and\;b_{i+1}<T\;and\;b_{i+2}<T\;\;\;i=1,2,\cdots ,r-3$$

where the first $k_{1}$ singular values correspond to effective signals. 

For two-dimensional GPR data $B\in R^{N\times L}$, the noise suppression method based on SVD of a window-length-optimized Hankel matrix can be summarized by the following steps:

1. Select the data of one trace (one-dimensional data) from two-dimensional GPR data and use the one-dimensional data to form a Hankel matrix with a certain window length by Equation (1).

2. Decompose the Hankel matrix by Equation (3) and compute the FRFCM of singular values by Equation (7).

3. Repeat steps 1 and 2 for different window lengths and obtain the optimal window length by Equation (8).

4. For the Hankel matrix with optimal window length, calculate the difference spectrum of singular values and obtain a threshold by Equations (9) and (10).

5. Select the demarcation point between singular values corresponding to effective signals and singular values corresponding to the noise by Equation (11).

6. Reconstruct the denoised Hankel matrix with singular values corresponding to effective signals by Equation (5) and obtain the denoised one-dimensional data by Equation (6).

7. Repeat steps 1–6 for all the traces and implement noise removal for two-dimensional GPR data.

## 3. Results and Discussion

A series of synthetic and real data is used to evaluate the proposed method. In addition, the performance of the proposed method is also compared with those of the conventional SVD method based on the local energy ratio rule and the wavelet transform method. The synthetic data are also generated by the “gprMax” simulator [31] based on the finite difference time domain (FDTD) method [33]. All the programs are executed on a 3.60 _GHz CPU and 32_GB memory computer.

### 3.1. Synthetic Example 1

**Figure 6:**
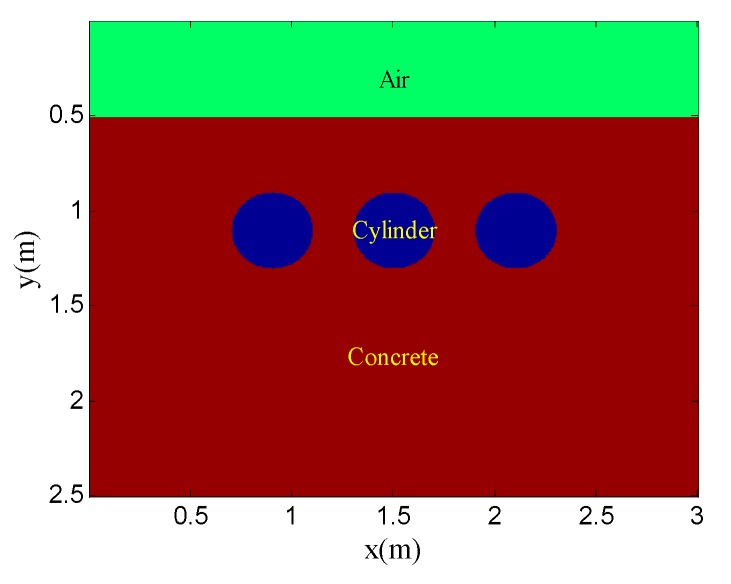
Geometry of the simulation model for point target detection.

**Figure 7:**
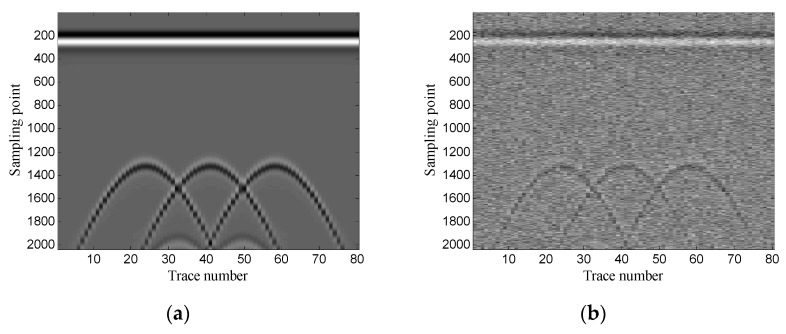
Synthetic GPR image: (**a**) original image; (**b**) noisy image.

The example shows the scenario of point target detection. Figure 6 shows the geometry of the simulation model. The targets are three perfect conductor metal cylinders with 0.4 m diameter and they are buried at the same depth of 0.6 m. The interval of the three targets is 0.6 m. The transmitting antenna is placed in the air layer and excited by a Ricker wavelet with a center frequency of 900 MHz. There are 80 traces in total and the trace interval is 0.035 m. The time window for each trace is 12 ns and each trace contains 2036 sampling points. Figure 7 shows the original GPR image and the noisy GPR image (SNR= −5.00 dB). 

**Figure 8:**
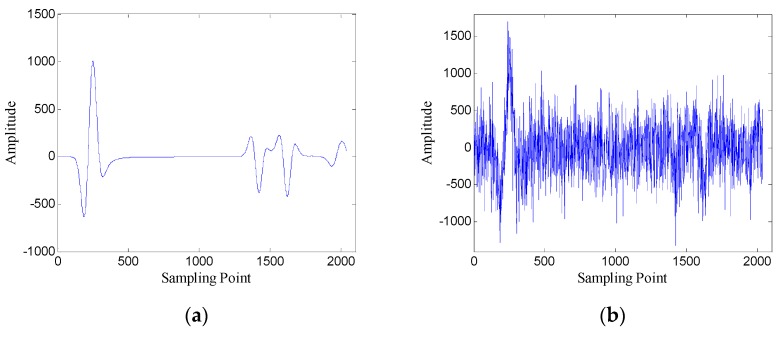
Data of trace 30: (**a**) original data; (**b**) noisy data (SNR = −4.62 dB).

First, the performance of the proposed method is analyzed using one-dimensional data. Figure 8 shows the data of trace 30.

**Figure 9:**
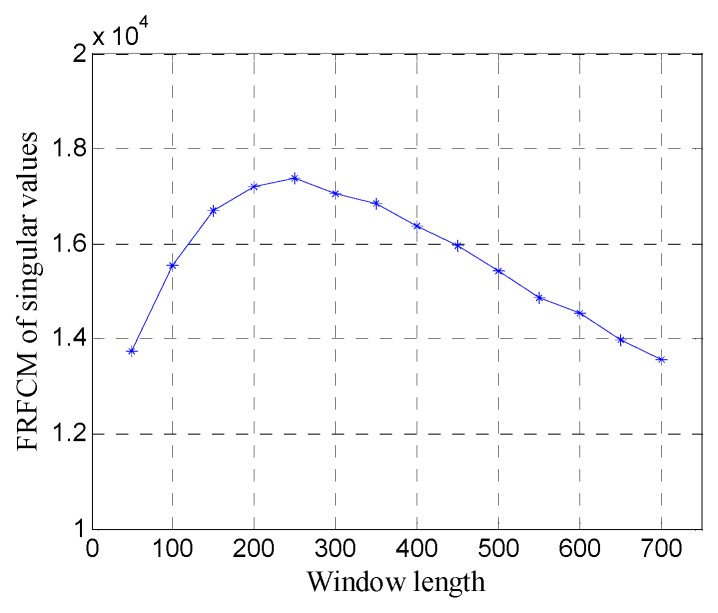
The fourth root of the fourth central moment (FRFCM) of singular values for Hankel matrices with different window lengths.

Figure 9 shows the FRFCM of singular values for Hankel matrices with different window lengths. It can be seen that when the window length increases, the FRFCM of singular values first increases and then decreases and reaches the maximum when the window length is 250. Therefore, the optimal window length for the Hankel matrix is 250.

According to the selection method of singular values, the demarcation point $k_{1}$ is 6. Then the Hankel matrix is reconstructed with the first 6 singular values, and the denoised data are recovered from the reconstructed Hankel matrix. 

**Figure 10:**
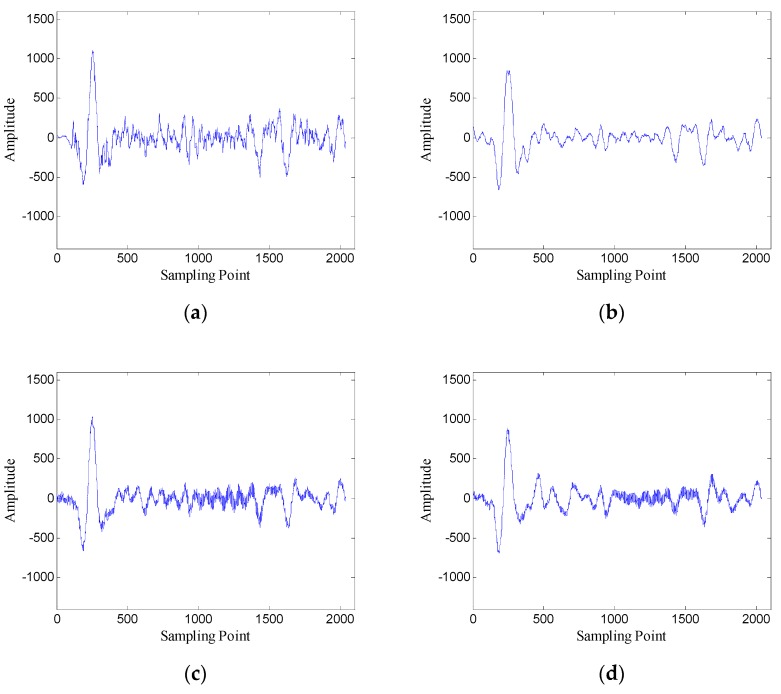
Denoised results with different window lengths for the data of trace 30: (**a**) *n* = 100 (SNR = 5.43 dB); (**b**) *n* = 250 (SNR = 7.45 dB); (**c**) *n* = 400 (SNR = 6.66 dB); (**d**) *n* = 700 (SNR = 5.19 dB).

In order to verify the performance of the window length optimization method, the denoised results with several different window lengths are shown in Figure 10. When the window length is 100, the denoised data contain many burrs; when the window length is 250, the denoised data are relatively smooth; when the window length is 400 and 700, the denoised data also contain some noise. The results preliminarily verify the effectiveness of the window length optimization method. 

**Figure 11:**
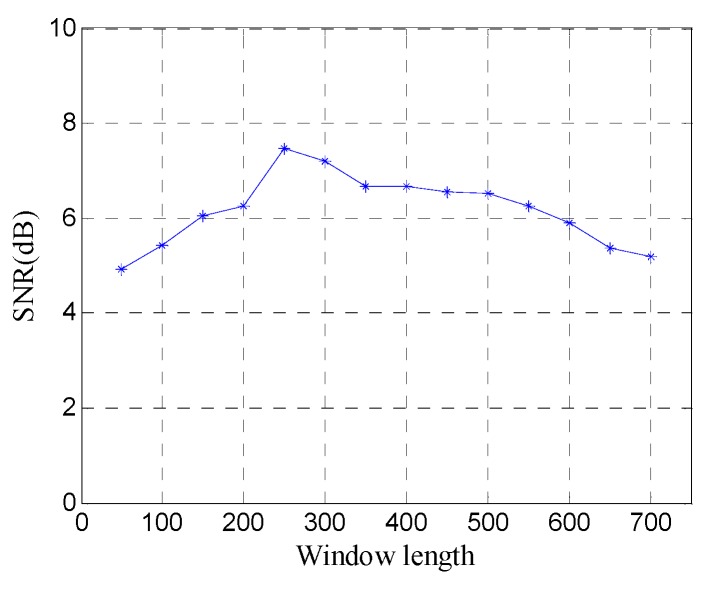
SNR of denoised data with different window lengths.

In order to quantitatively analyze the performance of the window length optimization method, the SNR of denoised data with different window lengths is shown in Figure 11. The SNR exhibits a fluctuation similar to the FRFCM of singular values, and reaches the maximum 7.45 dB at the optimal window length (*n*=250), which shows that the window length optimization method can obtain the best noise removal performance for SVD of the Hankel matrix.

**Figure 12:**
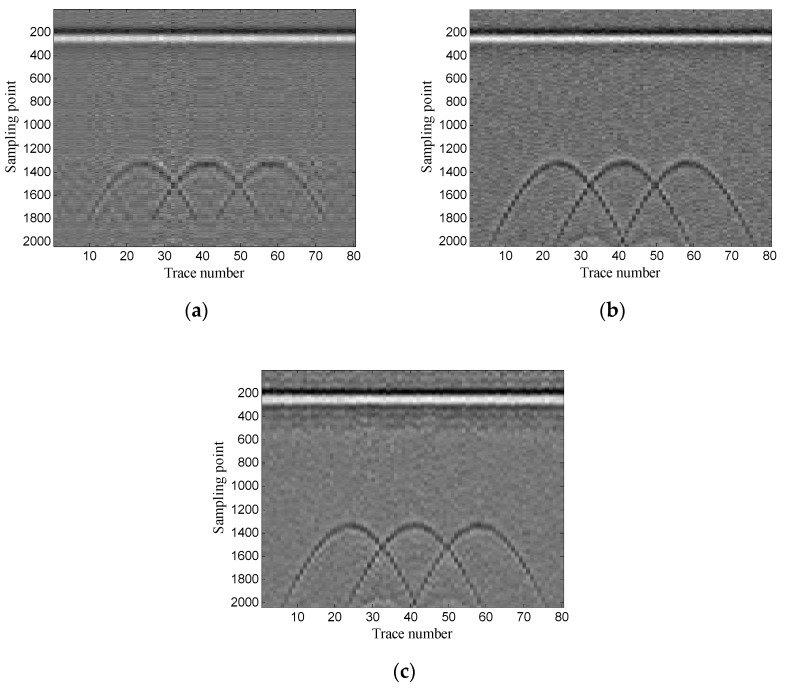
Denoised results of the three methods for a GPR image: (**a**) singular value decomposition (SVD) method based on the local energy ratio rule; (**b**) wavelet transform method; (**c**) proposed method.

Then, the performance of the proposed method is verified using two-dimensional data. In addition, the experimental results of the proposed method are compared with those of the conventional SVD method based on the local energy ratio rule and the wavelet transform method. Figure 12 shows the denoised results of the three methods. As shown in Figure 12a the conventional SVD method can remove noise, but it also removes some of the target signals. As shown in Figure 12b, the wavelet transform method retains complete target signals, but it also retains a small amount of noise. As shown in Figure 12c the proposed method can retain complete target signals while removing more noise. 

Table 1 lists the SNR, processing time, and the amount of RAM memory required for the three methods. As shown in Table 1, the proposed method yields a higher SNR than the other two methods, and it also needs more processing time and larger RAM memory than the other two methods due to the calculation of SVD of the Hankel matrix for each one-dimensional data. 

### 3.2. Synthetic Example 2

**Figure 13:**
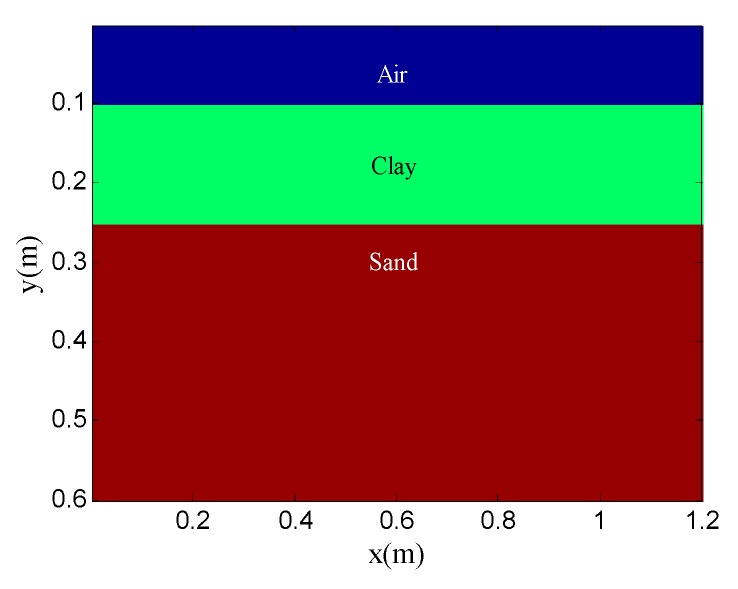
Geometry of the simulation model for layer detection.

**Figure 14:**
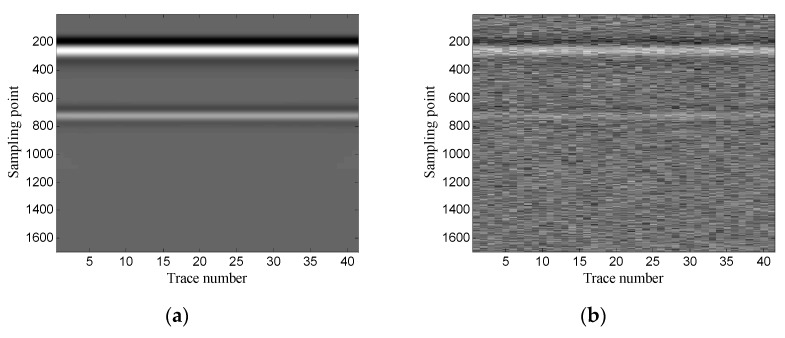
Synthetic GPR image: (**a**) original image; (**b**) noisy image.

The example shows the scenario of layer detection. Figure 13 shows the geometry of the simulation model. The model contains two layers: clay and sand. The transmitting antenna is placed in the air layer and excited by a Ricker wavelet with a center frequency of 900 MHz. There are 41 traces in total and the trace interval is 0.02 m. The time window for each trace is 10 ns and each trace contains 1696 sampling points. Figure 14 shows the original GPR image and the noisy GPR image (SNR= −5.00 dB).

**Figure 15:**
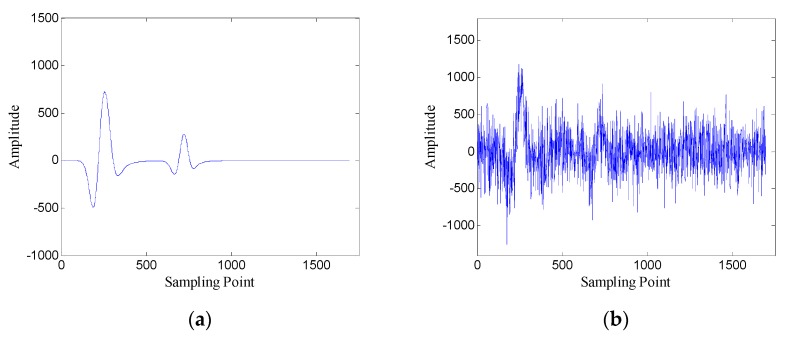
Data of trace 20: (**a**) original data; (**b**) noisy data (SNR = −5.12 dB).

**Figure 16:**
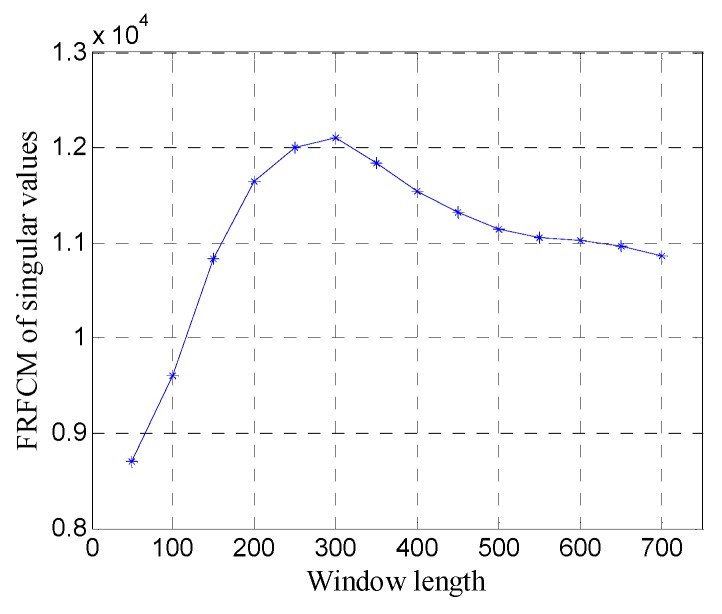
FRFCM of singular values for Hankel matrices with different window lengths.

First, the one-dimensional data are used to verify the performance of the proposed method. Figure 15 shows the data of trace 20. Figure 16 shows the FRFCM of singular values for Hankel matrices with different window lengths. Evidently, FRFCM reaches the maximum when the window length is 300. Therefore, the optimal window length for the Hankel matrix is 300. The demarcation point $k_{1}$ is set to 6 by the selection method of singular values. Then the Hankel matrix is reconstructed with the first 6 singular values, and the denoised data are recovered from the reconstructed Hankel matrix.

**Figure 17:**
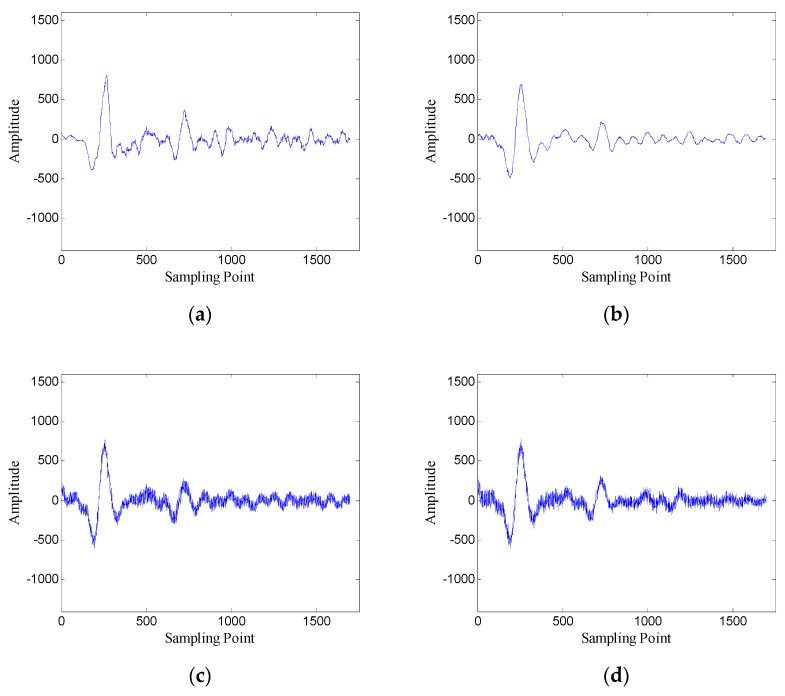
Denoised results with different window lengths for the data of trace 20: (**a**) *n* = 150 (SNR = 5.91 dB); (**b**) *n* = 300 (SNR = 7.86 dB); (**c**) *n* = 450 (SNR = 5.82 dB); (**d**) *n* = 600 (SNR = 5.40 dB).

The denoised results with several different window lengths are shown in Figure 17. As the figure shows, the optimal window length can obtain the best compromise between noise suppression and retaining effective signals.

**Figure 18:**
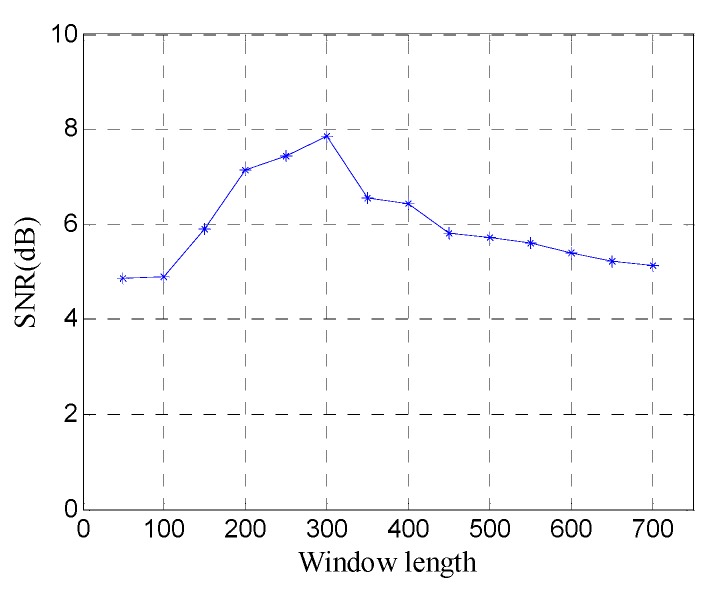
SNR of denoised data with different window lengths.

In order to quantitatively analyze the performance of the window length optimization method, the SNR of denoised data with different window lengths is shown in Figure 18. The results further show the window length optimization method can achieve the best noise removal performance for SVD of the Hankel matrix.

**Figure 19:**
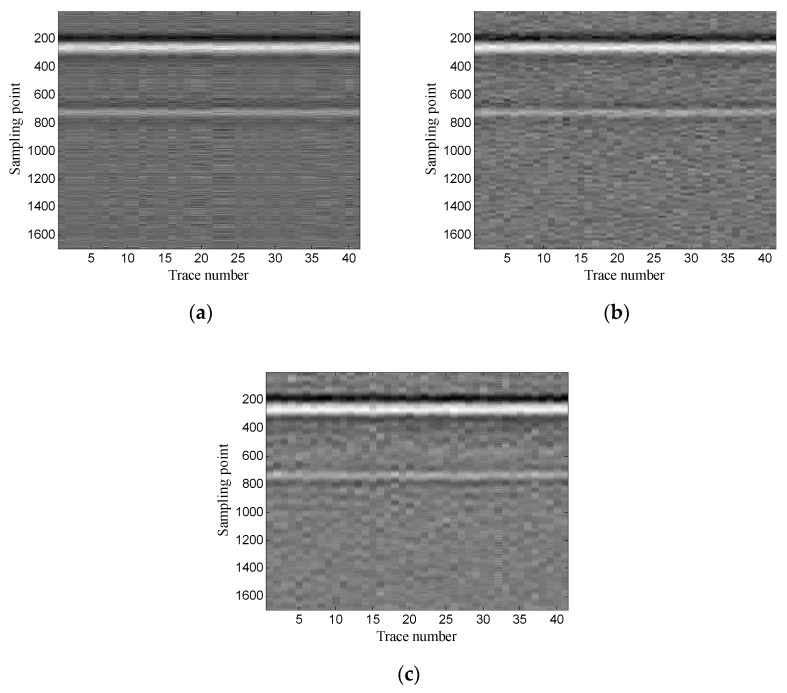
Denoised results of the three methods for a GPR image: (**a**) SVD method based on the local energy ratio rule; (**b**) wavelet transform method; (**c**) proposed method.

Then, the two-dimensional data are used to verify the performance of the proposed method. The experimental results of the proposed method are also compared with those of the conventional SVD method based on the local energy ratio rule and wavelet transform method. Figure 19 shows the denoised results of the three methods. As shown in Figure 19a, the layer signals are relatively weak, and some horizontal noise is also introduced. Figure 19b shows that the layer signals are obvious, but a small amount of noise is also retained; and Figure 19c shows that the layer signals are relatively strong, and the noise is also removed more thoroughly. 

Table 2 lists the SNR, processing time, and the amount of RAM memory required for the three methods. Table 2 also shows that the proposed method yields a higher SNR and consumes more memory space compared with the other two methods. 

### 3.3. Synthetic Example 3

**Figure 20:**
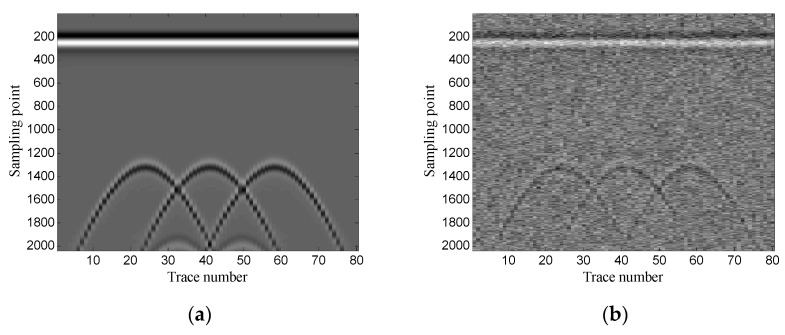
Synthetic GPR image: (**a**) original image; (**b**) noisy image.

In this section, the performance of the proposed method is investigated in the presence of correlated noise. This example uses the same original GPR image as synthetic example 1. The autocorrelation function of the noise is an exponential function and the correlation length of the noise is 10. Figure 20 shows the original GPR image and the noisy GPR image (SNR = −5.00 dB). 

**Figure 21:**
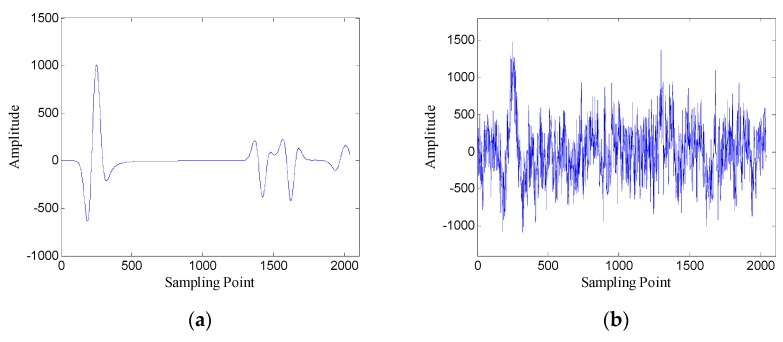
Data of trace 30: (**a**) original data; (**b**) noisy data (SNR = −4.55 dB).

**Figure 22:**
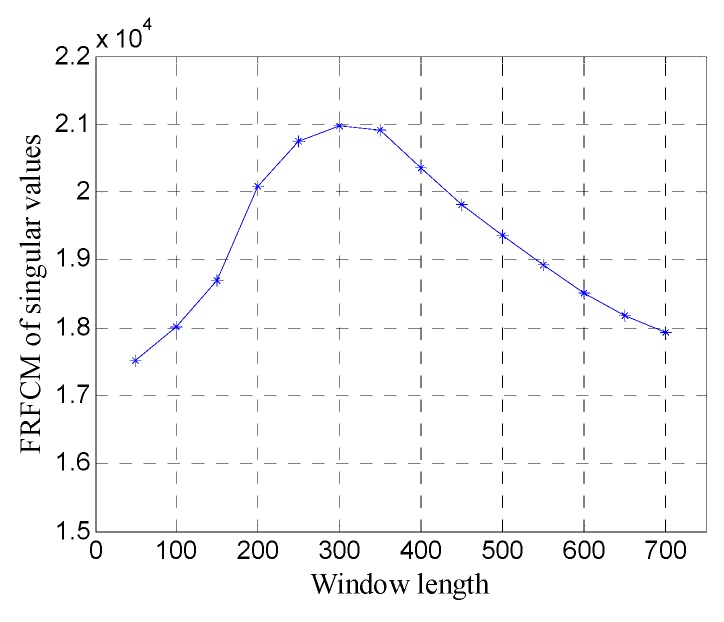
FRFCM of singular values for Hankel matrices with different window lengths.

First, the performance of the proposed method is analyzed using one-dimensional data. Figure 21 shows the data of trace 30. Figure 22 shows the FRFCM of singular values for Hankel matrices with different window lengths. In this case, it is evident that the value of FRFCM is greater than that in the case of white noise and the optimal window length for the Hankel matrix is 300. The Hankel matrix is reconstructed with the first eight singular values, and the denoised data are recovered from the reconstructed Hankel matrix. 

**Figure 23:**
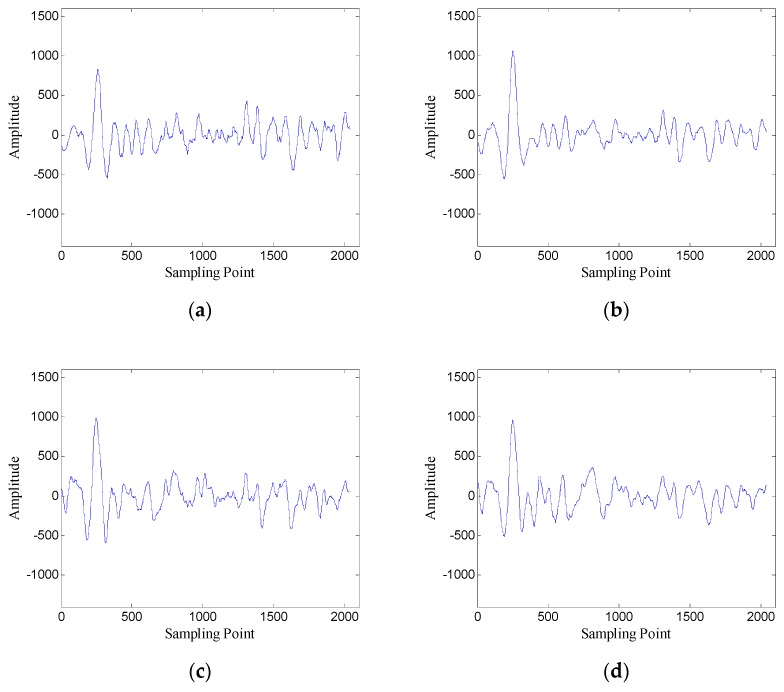
Denoised results with different window lengths for the data of trace 30: (**a**) *n* = 100 (SNR = 1.72 dB); (**b**) *n* = 300 (SNR = 4.31 dB); (**c**) *n* = 500 (SNR = 2.96 dB); (**d**) *n* = 650 (SNR = 2.39 dB).

Figure 23 shows the denoised results with several different window lengths. When the window length is 100, the denoised data contain some oscillating components; when the window length is 500 and 650, the denoised data also contain a lot of interference with large amplitude; when the window length is 300, the denoised data contain the least noise. The results confirm that the window length optimization method is also effective in the case of correlated noise. 

**Figure 24:**
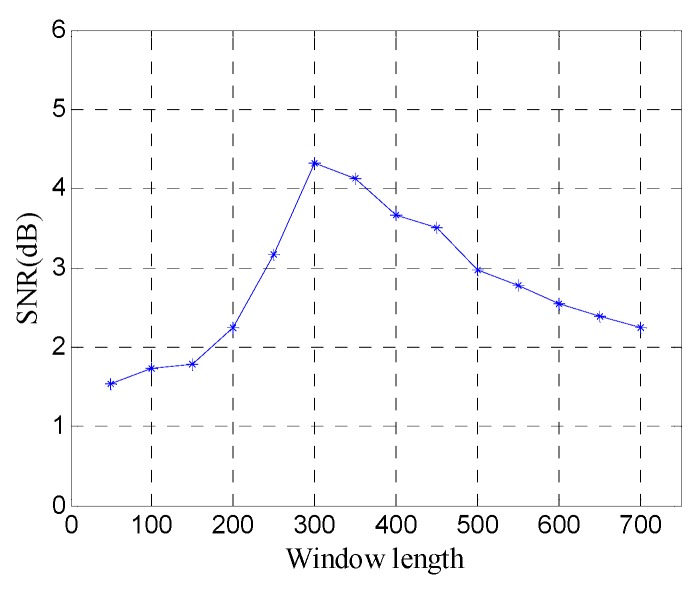
SNR of denoised data with different window lengths.

Figure 24 shows the SNR of denoised data with different window lengths. The results show that SVD of the Hankel matrix obtains the best noise removal performance at the optimal window length (*n* =300).

**Figure 25:**
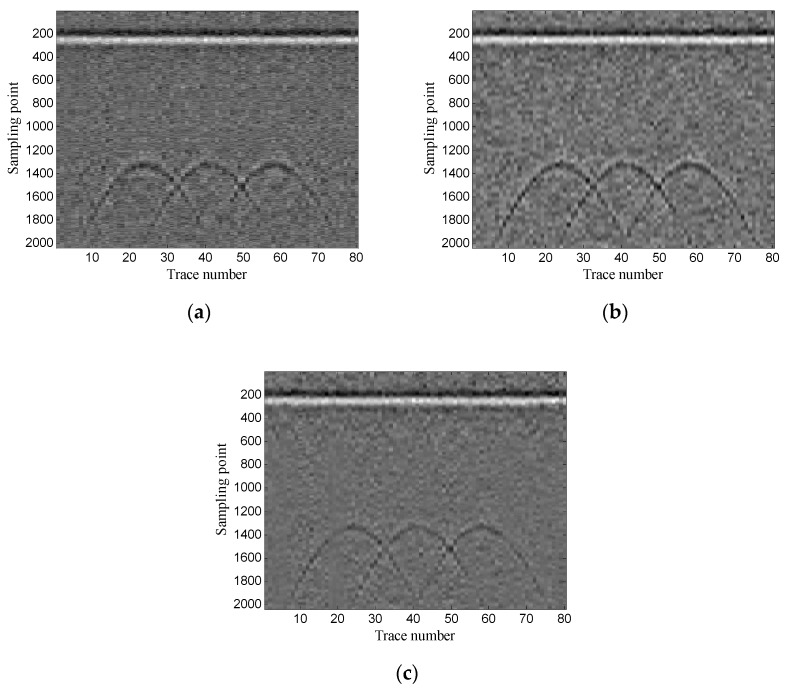
Denoised results of the three methods for a GPR image: (**a**) SVD method based on the local energy ratio rule; (**b**) wavelet transform method; (**c**) proposed method.

Then, the performance of the proposed method is verified using two-dimensional data. Moreover, the experimental results of the proposed method are compared with those of the conventional SVD method based on local energy ratio rule and the wavelet transform method. Figure 25 shows the denoised results of the three methods. As shown in Figure 25a the conventional SVD method removes some target signals while denoising. Figure 25b shows that the wavelet transform method also retains some noise while retaining target signals; and Figure 25c shows that the proposed method retains more target signals while removing more noise.

Table 3 lists the SNR, processing time, and the amount of RAM memory required for the three methods. Compared with the results of synthetic example 1, the SNR of the three methods all decreases due to the correlation of the noise. The proposed method yields an obviously higher SNR with an appropriate increase in processing time compared with the other two methods. In addition, the wavelet transform method requires larger RAM memory due to the correlation of the noise. 

### 3.4. Synthetic Example 4

**Figure 26:**
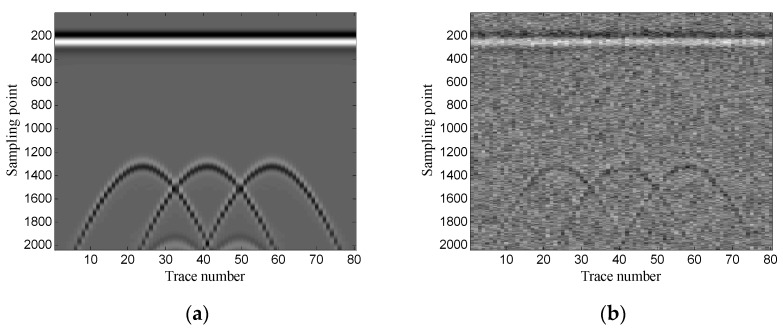
Synthetic GPR image: (**a**) original image; (**b**) noisy image.

In this section, the performance of the proposed method is also investigated in the presence of correlated noise. This example also uses the same original GPR image as synthetic example 1. The autocorrelation function of the noise is an exponential function and the correlation length of the noise is 20. Figure 26 shows the original GPR image and the noisy GPR image (SNR = −5.00 dB). 

**Figure 27:**
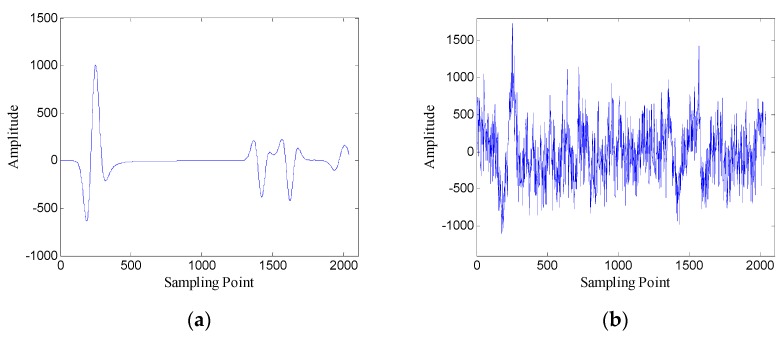
Data of trace 30: (**a**) original data; (**b**) noisy data (SNR = −4.49 dB).

**Figure 28:**
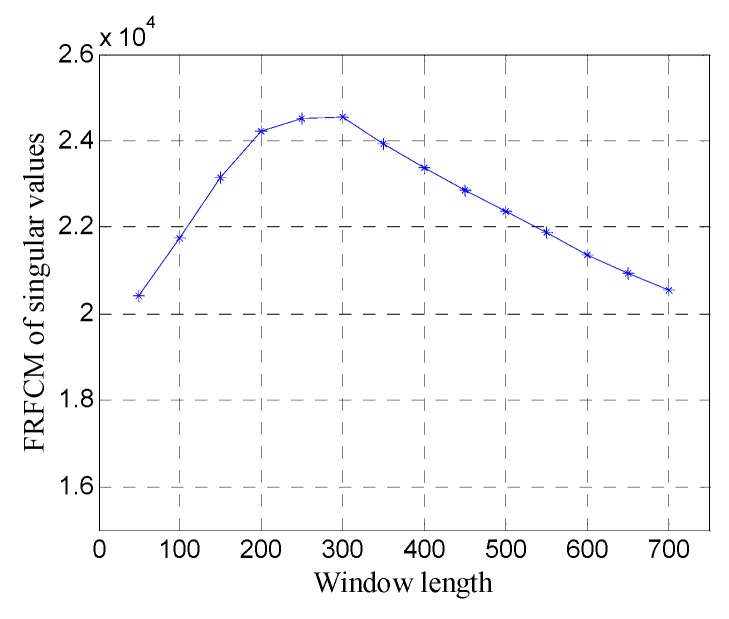
FRFCM of singular values for Hankel matrices with different window lengths.

First, the performance of the proposed method is analyzed using one-dimensional data. Figure 27 shows the data of trace 30. Figure 28 shows the FRFCM of singular values for Hankel matrices with different window lengths. Evidently, the correlation length of the noise increases, the value of FRFCM also increases, and the optimal window length for the Hankel matrix is 300. The Hankel matrix is reconstructed with the first nine singular values and the denoised data are recovered from the reconstructed Hankel matrix. 

**Figure 29:**
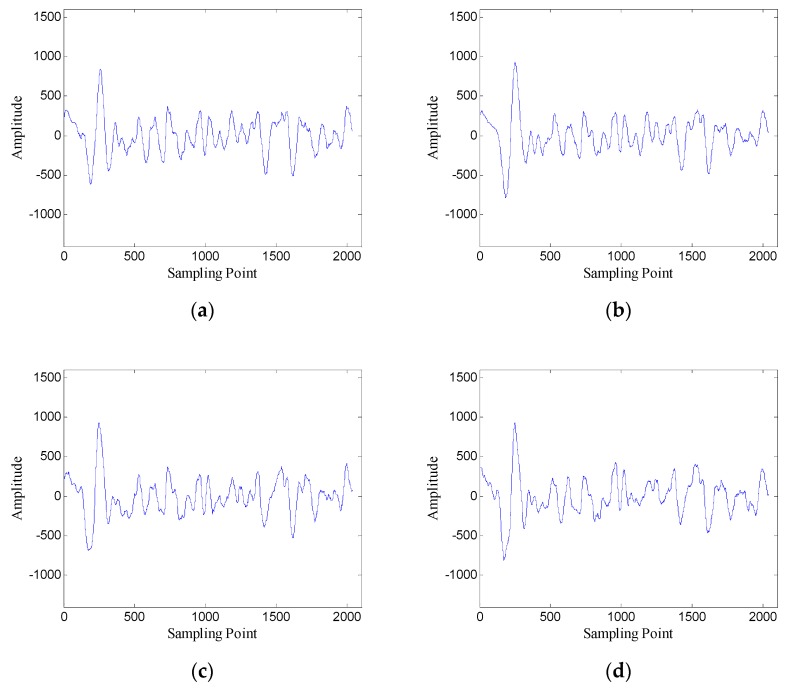
Denoised results with different window lengths for the data of trace 30: (**a**) *n* = 100 (SNR = 1.61 dB); (**b**) *n* = 300 (SNR = 3.02 dB); (**c**) *n* = 500 (SNR = 2.16 dB); (**d**) *n* = 650 (SNR = 1.66 dB).

Figure 29 shows the denoised results with several different window lengths. As the figure shows, denoised data of the optimal window length contain less noise than those of other window lengths.

**Figure 30:**
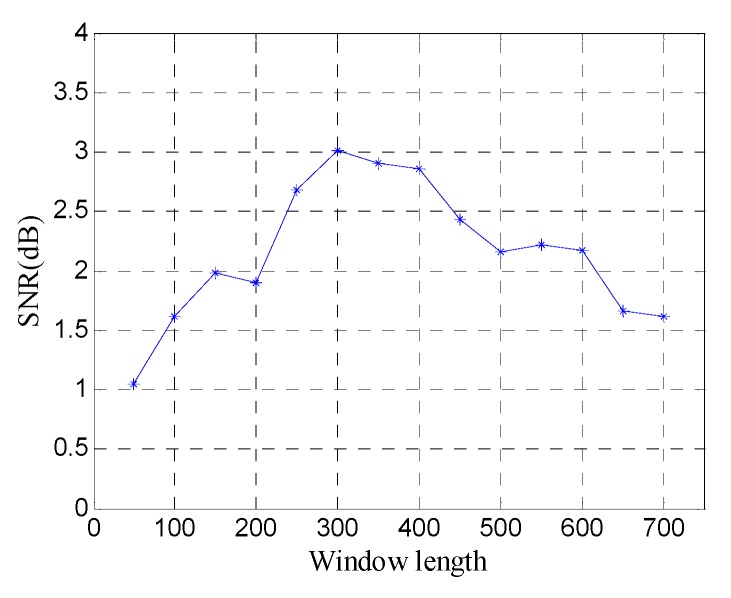
SNR of denoised data with different window lengths.

Figure 30 shows the SNR of denoised data with different window lengths. The SNR exhibits more fluctuation, and it reaches maximum at the optimal window length (*n* = 300).

**Figure 31:**
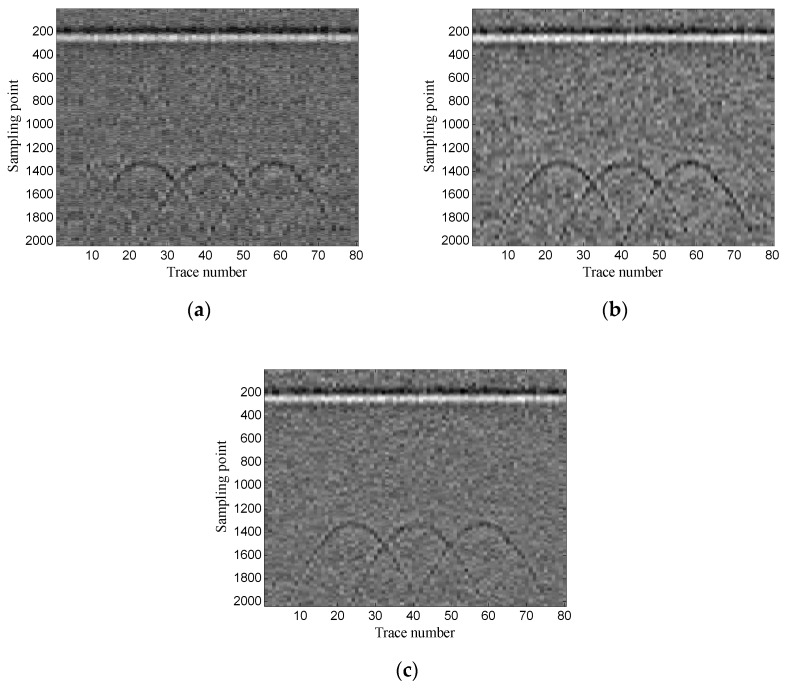
Denoised results of the three methods for a GPR image: (**a**) SVD method based on the local energy ratio rule; (**b**) wavelet transform method; (**c**) proposed method.

Then, the performance of the proposed method is verified using two-dimensional data. The experimental results of the proposed method are also compared with those of the conventional SVD method based on the local energy ratio rule and the wavelet transform method. Figure 31 shows the denoised results of the three methods. As shown in Figure 31, the conventional SVD method loses a lot of target signals; the wavelet transform method also retains a lot of noise while retaining target signals; the proposed method achieves a good compromise between retaining target signals and removing the noise.

Table 4 lists the SNR, processing time, and the amount of RAM memory required for the three methods. Compared with the results of synthetic example 3, the increase of the correlation length of the noise obviously degrades the SNR of the three methods. The proposed method also achieves a higher SNR at the cost of the increasing processing time and more memory space compared with the other two methods. 

### 3.5. Field Measurements 1

**Figure 32:**
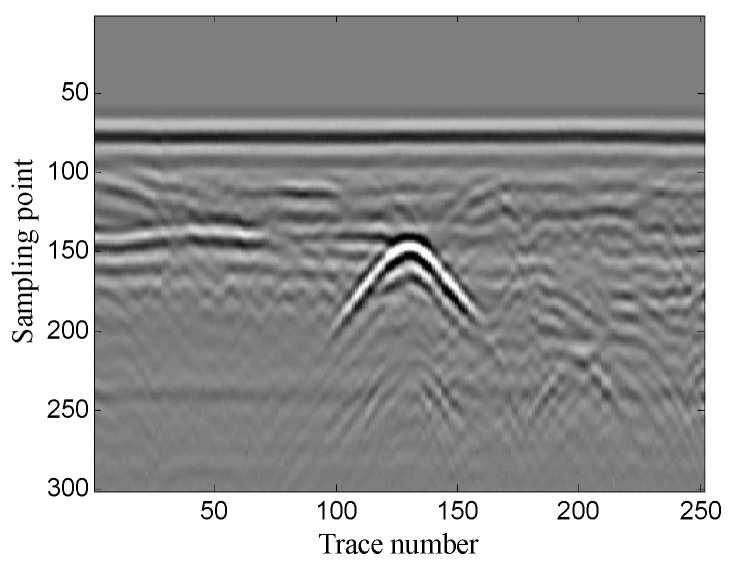
Real GPR image of pipeline detection.

The example shows the scenario of pipeline detection. The antenna center frequency is 400 MHz. There are 251 traces in total and each trace contains 301 sampling points. Figure 32 shows the original noisy GPR image. As the figure shows, there is a lot of noise around the target hyperbolic signals, which affects the target detection.

**Figure 33:**
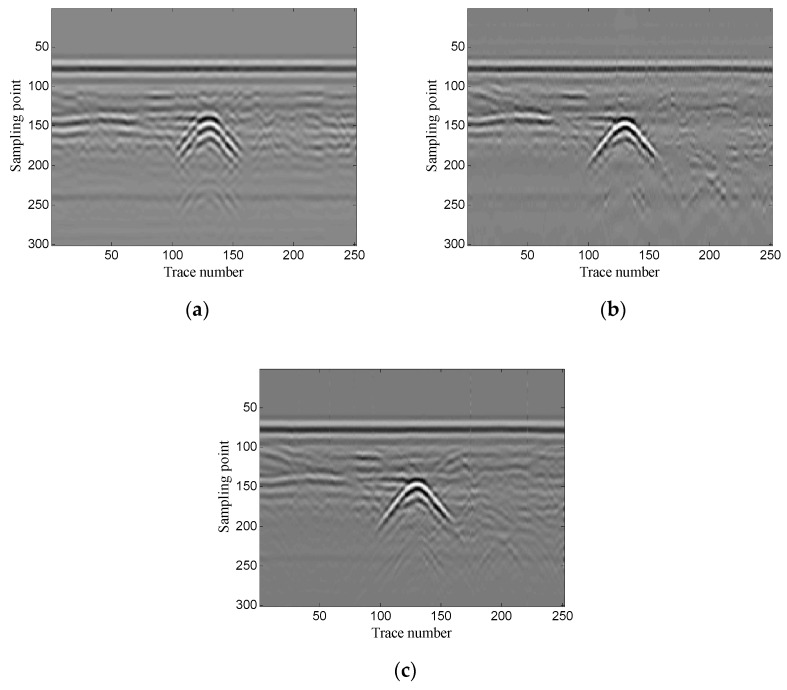
Denoised results of the three methods for a real GPR image: (**a**) SVD method based on the local energy ratio rule; (**b**) wavelet transform method; (**c**) proposed method.

The optimal window length for the Hankel matrix is 90. The denoised results of the three methods are shown in Figure 33. As shown in Figure 33a the conventional SVD method removes some of the noise, but it generates some false target hyperbolic signals. Figure 33b shows that the wavelet transform method removes most of the noise and retains complete target signals, but it introduces a small amount of vertical noise; Figure 33c shows that the proposed method removes most of the noise and preserves complete target signals without introducing any other signals. The results show that the proposed method achieves better noise removal performance than the other two methods and helps to detect the pipeline accurately.

The processing time of the conventional SVD method, the wavelet transform method, and the proposed method is 0.45 s, 1.32 s, and 1.77 s, respectively. 

### 3.6. Field Measurements 2

**Figure 34:**
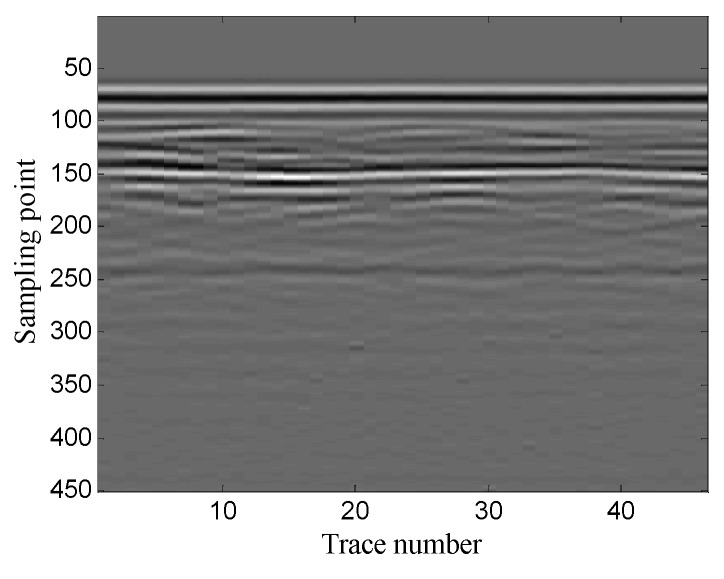
Real GPR image of road layer detection.

The example shows the scenario of road layer detection. The antenna center frequency is 400 MHz. There are 46 traces in total and each trace contains 450 sampling points. Figure 34 shows the original noisy GPR image. As the figure shows, there is some horizontal noise around the layer signals, which interferences with the layer recognition.

**Figure 35:**
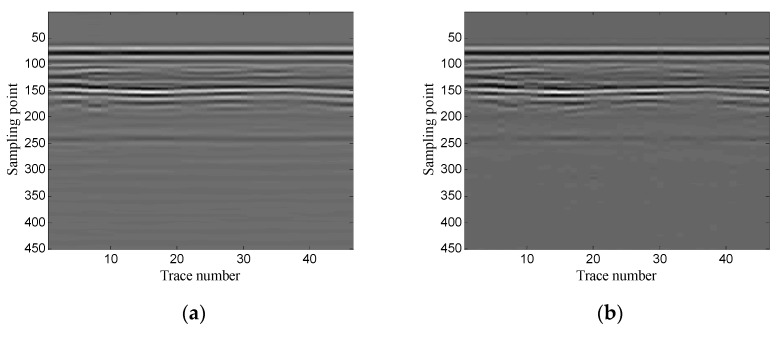

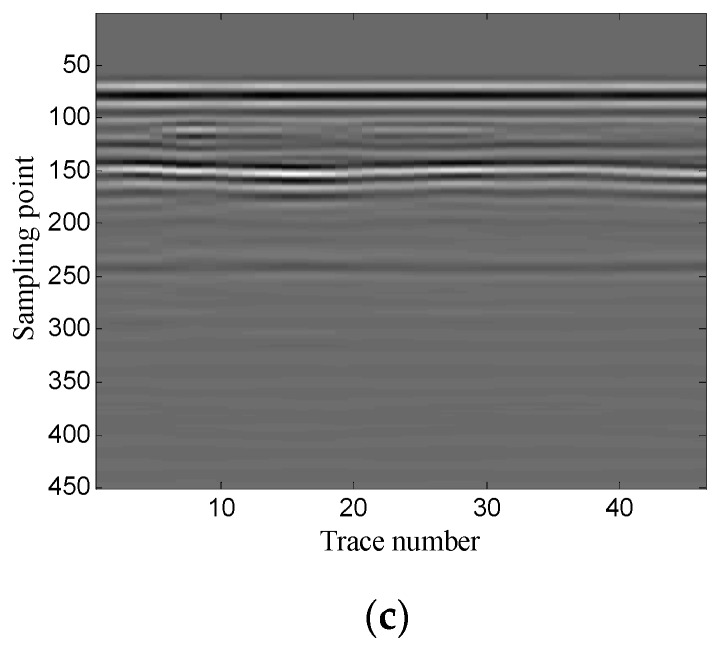
Denoised results of the three methods for a real GPR image: (**a**) SVD method based on the local energy ratio rule; (**b**) wavelet transform method; (**c**) proposed method.

The optimal window length for the Hankel matrix is 110. The denoised results of the three methods are shown in Figure 35. As shown in Figure 35a, the conventional SVD method removes some of the noise, but it still retains some noise between the 80th and the 150th sampling points. Figure 35b shows that the wavelet transform method retains a small amount of noise between the 80th and the 150th sampling points, but it removes part of the layer signals near the 240th sampling point; Figure 35c shows that the proposed method removes most of the noise, and it retains the layer signals completely. The results show that the proposed method obtains the best noise removal performance and provides the best profile for layer detection. 

The processing time of the conventional SVD method, the wavelet transform method, and the proposed method is 0.14 s, 0.47 s, and 0.67 s, respectively. 

## 4. Conclusions

In this paper, a method based on SVD of a window-length-optimized Hankel matrix is proposed to improve the noise suppression performance for GPR data. The fourth root of the fourth central moment of singular values is used to determine the window length of the Hankel matrix, which provides a solution to optimize the size of the Hankel matrix. Then, the difference spectrum of singular values is used to construct a threshold, which provides a solution to select singular values corresponding to effective signals.

The proposed method is verified by series of synthetic and practical data. The results show the proposed method can obtain the best noise removal performance for both white noise and correlated noise. The proposed method also achieves better denoising performance than the conventional SVD method based on the local energy ratio rule and wavelet transform method at the cost of the appropriate increases in processing time and memory space. Future work will investigate more efficient solutions to optimize SVD of the Hankel matrix to further improve noise removal performance.

## Figures and Tables

**Table 1:** Results of the three methods.

Method	SNR (dB)	Processing Time (s)	Amount of RAM Memory (MB)
SVD method based on local energy ratio rule	4.23	1.9	71
Wavelet transform method	7.08	2.31	39
Proposed method	7.55	4.17	99

**Table 2:** Results of the three methods.

Method	SNR (dB)	Processing Time (s)	Amount of RAM Memory (MB)
SVD method based on local energy ratio rule	5.6	0.78	48
Wavelet transform method	7.03	0.92	17
Proposed method	7.42	1.43	53

**Table 3:** Results of the three methods.

Method	SNR (dB)	Processing Time (s)	Amount of RAM Memory (MB)
SVD method based on local energy ratio rule	0.94	2.16	75
Wavelet transform method	2.1	2.41	51
Proposed method	4.21	4.19	101

**Table 4:** Results of the three methods.

Method	SNR (dB)	Processing Time (s)	Amount of RAM Memory (MB)
SVD method based on local energy ratio rule	0.02	1.94	74
Wavelet transform method	0.59	2.64	53
Proposed method	2.05	4.15	102
